# Cross-cultural adaptation and exploratory factor analysis of the Person-centred Practice Inventory - Staff (PCPI-S) questionnaire among Malaysian primary healthcare providers

**DOI:** 10.1186/s12913-020-06012-9

**Published:** 2021-01-07

**Authors:** Nur Zahirah Balqis-Ali, Pui San Saw, Anis Syakira Jailani, Weng Hong Fun, Noridah Mohd Saleh, Tengku Putri Zaharah Tengku Bahanuddin, Sondi Sararaks, Shaun Wen Huey Lee

**Affiliations:** 1 Centre for Health Outcomes Research, Institute for Health Systems Research, National Institutes of Health, Block B2, No. 1, Jalan Setia Murni U13/52, Seksyen U13 Bandar Setia Alam, 40170 Shah Alam, Selangor Malaysia; 2 School of Pharmacy, Monash University Malaysia, Jalan Lagoon Selatan, 47500 Bandar Sunway, Selangor Malaysia; 3 Jeffrey Cheah School of Medicine and Health Sciences, Monash University Malaysia, Jalan Lagoon Selatan, 47500 Bandar Sunway, Selangor Malaysia; 4 Family Health Development Division, Ministry of Health Malaysia, 62590 Putrajaya, Malaysia; 5 School of Pharmacy, Taylor’s University Lakeside Campus, Jalan Taylors, 47500 Subang Jaya, Selangor Malaysia

**Keywords:** Person-centred, Primary care, Healthcare provider, Cross-cultural adaptation, Malaysia, PCPI-S, Questionnaire validation

## Abstract

**Background:**

The Person-centred Practice Inventory-Staff (PCPI-S) instrument was developed to measure healthcare providers’ perception towards their person-centred practice. The study aimed to explore the influence of culture, context, language and local practice towards the PCPI-S instrument adaptation process for use among public primary care healthcare providers in Malaysia.

**Methods:**

The original PCPI-S was reviewed and adapted for cultural suitability by an expert committee to ensure conceptual and item equivalence. The instrument was subsequently translated into the local Malay language using the forward-backward translation by two independent native speakers, and modified following pre-tests involving cognitive debriefing interviews. The psychometric properties of the corresponding instrument were determined by assessing the internal consistency, test-retest reliability, and correlation of the instrument, while the underlying structure was analysed using exploratory factor analysis.

**Results:**

Review by expert committee found items applicable to local context. Pre-tests on the translated instrument found multiple domains and questions were misinterpreted. Many translations were heavily influenced by culture, context, and language discrepancies. Results of the subsequent pilot study found mean scores for all items ranged from 2.92 to 4.39. Notable ceiling effects were found. Internal consistency was high (Cronbach’s alpha > 0.9). Exploratory factor analysis found formation of 11 components as opposed to the original 17 constructs.

**Conclusion:**

The results of this study provide evidence regarding the reliability and underlying structure of the PCPI-S instrument with regard to primary care practice. Culture, context, language and local practice heavily influenced the adaptation as well as interpretation of the underlying structure and should be given emphasis when translating person-centred into practice.

## Background

In many healthcare systems and organizations, a person-centred care (PCC) model is seen as a possible solution to improve health system performance to meet the growing demand for improved patient experience and health outcomes. It addresses health and social needs by catering to individual preferences and values, thus actively involving consumers and carers in care decisions and planning [1]. As such, person-centred care shifts the conventional medically dominated, disease orientated, fragmented care towards one that is collaborative and relationship focused. This can be explained by the theoretical framework of PCC by McCormack and McCance which emphasized the therapeutic relationship between healthcare providers and service users [2]. The therapeutic relationship is promoted by values of respect for the person, individual’s right to self-determination, mutual respect and understanding. This would involve working with the individual alongside health professionals to develop appropriate solutions, and for the individual to take responsibility for their own health and guide clinical decisions. In Malaysia, this concept has been embraced as a principle embedded in the national health goal since 2006 [3–5]. To achieve this, healthcare providers and the workplace are required to embrace a person-centred culture and work collaboratively with patients to improve their health outcomes [6]. Given the evolving concept of person-centred care, an understanding of the various facets of person-centred care, and how they are perceived, are fundamental for policymakers to operationalize this concept.

The Person-centred Practice Inventory-Staff (PCPI-S) questionnaire was originally developed by Slater, McCance and McCormack to examine how healthcare staff perceived their person-centred practice, with the aim that this allows health care teams to translate evidence informed action into an actionable person-centred culture [7]. The PCPI-S is a 59-item questionnaire drawn from 17 constructs, addressing three distinct domains: attributes of healthcare providers, context in which care is delivered and extent of delivering care. The original tool was validated among nursing staff from eight acute hospital settings [7]. Since then, the questionnaire has been translated and used in Norway, which found the tool to be valid with an acceptable goodness of fit when used among nurses [8].

However, little is known if the PCPI-S questionnaire psychometric quality is maintained when used in a different geographical setting, among diverse healthcare provider categories such as physicians and pharmacists or in a primary care context. While there are studies describing the process of cultural adaptation and validation of various health related tools, limited studies explore and describe in-depth how culture and language influence the adaptation process, and what measures or approach should be put in place to address the complexity and challenges that occur during the adaptation process [8–10]. This is important as a poorly translated and adapted tool will ultimately lead to an instrument that is not equivalent to the original version, limiting its comparability as well as validity [11, 12].

The aim of this study was to translate, cross-culturally adapt and conduct exploratory factor analyses of the PCPI-S among multidisciplinary healthcare providers in primary care in Malaysia.

## Methods

The study is part of a comprehensive study evaluating person-centredness among healthcare providers in Malaysian primary care clinics (NMRR-18-309-40447). The methodology of this study has been published previously [13]. This study was conducted in two phases; the first Phase I which was a cross-cultural adaptation and translation of PCPI-S tool and the second phase, which was the psychometric evaluation of the PCPI tool.

In phase I, the PCPI-S questionnaire was reviewed by an expert committee consisting 8 primary care experts (3 policymakers, 1 public health specialist and 4 primary healthcare providers) to verify practicality of all items to local context. It was subsequently translated into Malay language using the forward-backward translation method by two independent bilingual speaking individuals. The emphasis in the translation was on conceptual and cultural equivalence, instead of linguistic accuracy. Discrepancies were discussed with all team members to achieve consensus. The instrument was pre-tested among the nine primary healthcare provider categories selected from four public primary care clinics. During the pre-tests, recruited respondents completed the bilingual PCPI-S questionnaire, followed by a cognitive debriefing session carried out by authors (NZB, PSS, ASJ, TPZ). Cognitive debriefing interviews were conducted to identify plausible explanations for any problems observed, hence allowing further qualitative analysis of difficulties encountered.

During cognitive debriefing, respondents from each clinic were divided into groups of 5 and interviewed for their understanding, acceptability as well as emotional impact of each item in order to detect confusing or misleading items/terms. The techniques ‘thinking aloud’, ‘probing’, and ‘observation of respondents’ behaviour’ were used to gauge respondents’ ability to comprehend all items [14, 15]. Responses were coded into one of the themes identified using a standardized behaviour coding form used to systematically identify questions requiring revision [16, 17]. The codes were: (1) request for clarification, (2) answer with uncertainty, (3) disagree with terms/sentences used, (4) do not know or wrong interpretation, (5) not applicable or non-response and (6) bilingual differences. When any item received a higher number of codes, further probing was warranted. In-depth exploration of these items helped clarify comprehension problems. It also allowed progress of the questionnaire improvement to be tracked systematically.

In phase II, the instrument was completed by public primary care healthcare providers from an urban state in Malaysia. Upon receiving organizational approval, printed questionnaires were distributed to eligible respondents from the nine healthcare providers category; family medicine specialists, medical doctors, pharmacists, medical assistants, nurses, occupational therapists, physiotherapists, dietitians, and nutritionists of any duration of work in the clinics. Healthcare providers who were unavailable during data collection were excluded from the study. All responses were returned in an opaque sealed envelope to ensure privacy and confidentiality. A minimum of 300 respondents were targeted to achieve a minimum of 5:1 ratio per item for factor analyses [18]. Data collection was conducted over 3 months for instrument adaptation and subsequently 2 months for psychometric evaluation, offering all respondents the opportunity to complete the questionnaire.

### Statistical analysis

The instrument was assessed for its psychometric properties, including the items’ floor and ceiling effects evaluated at 15% acceptance level [19]. Test-retest reliability and internal consistency was determined respectively using intraclass correlation coefficient and Cronbach’s alpha. Multicollinearity and sampling adequacy were done using Bartlett’s test of sphericity and Kaiser-Meyer-Olkin criterion (≥ 0.50) [20]. The factors were examined using Eigenvalue> = 1 (Kaiser criterion) and scree plot. The Exploratory Factor Analysis (EFA) was performed using the extraction method of Principal Component with Varimax (Variation Maximization) Rotation [20]. Cross loadings were examined and small coefficients below 0.4 suppressed. The percentage of variance explained was considered acceptable if it exceeded 60% [21–25]. All responses were included in the analysis as responses were randomly missing with negligible impact upon removal. All analyses were performed in SPSS version 21 (IBM Corp, Armonyx).

## Results

### Phase I

#### Expert review and questionnaire translation

A team of 8 primary care experts reviewed the PCPI-S instrument to assess items relevance and acceptance for use in Malaysia. No items were dropped. The complexity and singularity of the PCC framework directly correlates to the difficulty of translation. Minor modifications were made on the terminology used and sentence structure to reflect a culturally, semantically and regionally appropriate questionnaire.

#### Demographic background of pre-test respondents

Respondents (*n* = 194) were recruited from 20 primary care clinics in 4 major states in Malaysia over five rounds of pre-tests; with 4 clinics involved in each round. The mean of respondents’ service years was 10.9 ± 6.6 (0.2–31 years). Majority of the respondents were nurses (*n* = 82, 42%). Other sociodemographic characteristics are presented in Table 1.

**Table 1:** Pre-test respondents (*n* = 194) characteristics and clinic types

Participants (*n =* 194)	Pre-test 1 (*n* = 40)	Pre-test 2 (*n* = 39)	Pre-test 3 (*n =* 40)	Pre-test 4 (*n* = 45)	Pre-test 5 (*n* = 30)
**Profession**					
Family medicine specialist (*n =* 4)	1	0	2	0	1
Medical officer (*n =* 26)	7	8	5	2	4
Pharmacist (*n* = 27)	9	4	5	4	5
Nurse (*n =* 82)	14	16	15	24	13
Medical assistant (*n =* 32)	8	6	6	8	4
Physiotherapist (*n =* 7)	1	1	1	3	1
Occupational therapist (*n =* 7)	0	2	1	3	1
Nutritionist and dietician (*n* = 5)	0	1	2	1	1
Others (lab technician and assistant pharmacist) (*n =* 4)	0	1	3	0	0
**Clinic size (patient attendance/day)**					
Type 1 (> 800) (*n =* 1)	0	0	0	1	0
Type II (500–799) (*n =* 3)	1	0	1	0	1
Type III (300–499) (*n =* 11)	3	2	2	3	1
Type IV (150–299) (*n =* 5)	0	2	1	0	2

#### Behaviour coding

**Fig. 1:**
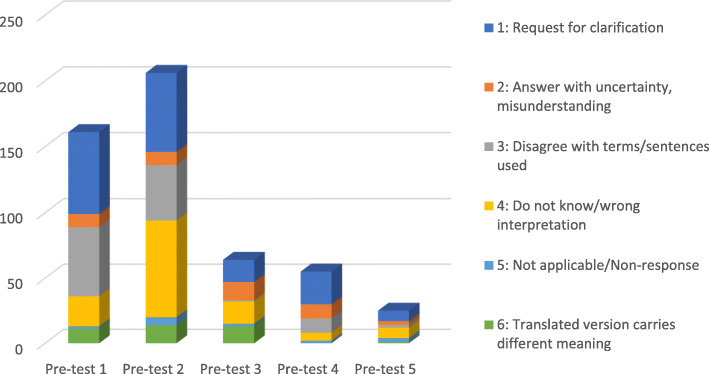
Distribution of behaviour codes indicating comprehension problems across five pre-tests

Participants required approximately 20 min to complete the questionnaire. During the cognitive debriefing session, respondents were also asked 22 specific probing questions, developed by the authors (Appendix 1). Issues indicating comprehension problems across the pre-tests is shown in Fig. 1.

Most of the modifications focused on improving individual translations in each item. For example, 64 respondents requested for clarification (Code 1) across all the 59 items in the questionnaire, necessitating substantial questionnaire modification. These can be broadly categorised into seven areas (Table 2): (1) words/phrases which were ambiguous, (2) synonyms, (3) grammatical meaning, (4) homonyms, (5) compound words, (6) unfamiliarity with subject experiences, and (7) words bearing emotional weight and action words. One-quarter of respondents requested clarification on the item “I continuously look for opportunities to improve the care experiences”. Reconciled comments revealed that respondents were unsure of the recipient of the care experiences in the question. Thus, examples were provided in the revised question which now read: “I continuously look for opportunities to improve the care experiences (example patient, family members, healthcare provider)”.

**Table 2:** Examples of revisions made to address comprehension problems

Main issues	Problems encountered	Item example	Action taken
Require further explanation	- Ambiguous - Grammatical meaning - Multiple meaning	(A1) I have the necessary skills to negotiate care options Translated versions were repeatedly misinterpreted despite terminology change and sentence restructuring.	Exemplar scenario added
Cultural discrepancy	- Multiple meaning	(D13) I take my time to explore why I react as I do in certain situations Translated term for “to explore why I react” *(“menilai semula”)* was negatively interpreted as reflection of avoidable mistakes and replaced with “positive self-reflection” (*“muhasabah”)*	Terminology replaced
	- Words bearing emotional weight and action word	(E17) I challenge colleagues when their practice is inconsistent with our team’s shared values and beliefs All possible terms to translate “challenge” were negatively perceived and deemed culturally rude. The original translation (“*mempertikai*”) was retained.	No modification
Language discrepancy	- Words bearing emotional weight and action word	(C8) I strive to deliver high quality care to people The translated phrase for the word ‘strive’ *(“berusaha”)* did not capture the intensity of enforcing greater effort. Word expanded to emphasize greater effort (“*berusaha dengan gigih*”).	Expansion of phrase
	- Compound words - Multiple meaning	(I29) The contribution of colleagues is recognized and acknowledged “Recognised and acknowledged” was translated to a single word “appreciated” (“*dihargai*”), instead of unnecessarily using 2 words (“*dikenalpasti dan diambilmaklum*”) which carried no additional meaning in Malay.	Replaced by single word
	- No similar word - Unfamiliarity with subject experience	(F20) I am able to make the case when skill mix falls below acceptable levels “S*kill-mix*” has no equivalent and meaningful term in Malay and was retained in English. The definition was added for reference.	Definition added
Require redirection	- Complex sentence	(M44) I integrate my knowledge of the person into care delivery Highlighting certain words in “I integrate my knowledge **of the person** into care delivery” was to emphasise and shift respondents’ focus from the knowledge about “the care and treatment for the person” to knowledge about “a person influencing care and treatment”.	Some words highlighted

Modifications (such as sentence restructuring) were made to improve discrepancy from cultural and language aspects. In items E17 and J35, the translated term for the word “*challenge*” in Malay, *“mempertikai”* appeared too intimidating for the respondents; suggesting an expression of sharp disapproval or criticism of someone. Comments reconciled from respondents viewed “challenging someone” as a negative and unacceptable practice in the local culture. Instead, respondents suggested alternative (translated) terms such as “*negotiate*”, *“rebuke”, “advice*” or “*offer*” but were not accepted in the adapted questionnaire as the authors felt that it does not reflect the intended meaning of “*stimulating or triggering by the way of disputing someone*”. The term *“mempertikai”* was therefore retained.

Overall, respondents seemed to be confused with the term “*person*” in the questionnaire and wanted clarification whether it was referring to a patient. They expressed that they were more familiar with the *‘patient-centred care’* term. Following this, the definition of person-centred care was included as an introductory statement to describe the person as the patient, carer/family member and healthcare provider.

A detailed listing of issues and solutions are presented in Appendix 2 and Appendix 3.

#### Rating scale

Respondents were observed to rate highly on all constructs of the questionnaire (mean score between 3.9 and 4.3) in the first 2 pre-tests. Cognitive debriefing revealed inaccurate ratings as respondents misconstrued the meaning of the “agree” scale as a personal opinion of “what should be carried out”, instead of “what is currently practised”. Furthermore, respondents revealed it was difficult to select *“disagree”* as it implied that they object to the ideas being proposed, or they were not competent in doing their work.

**Fig. 2:**
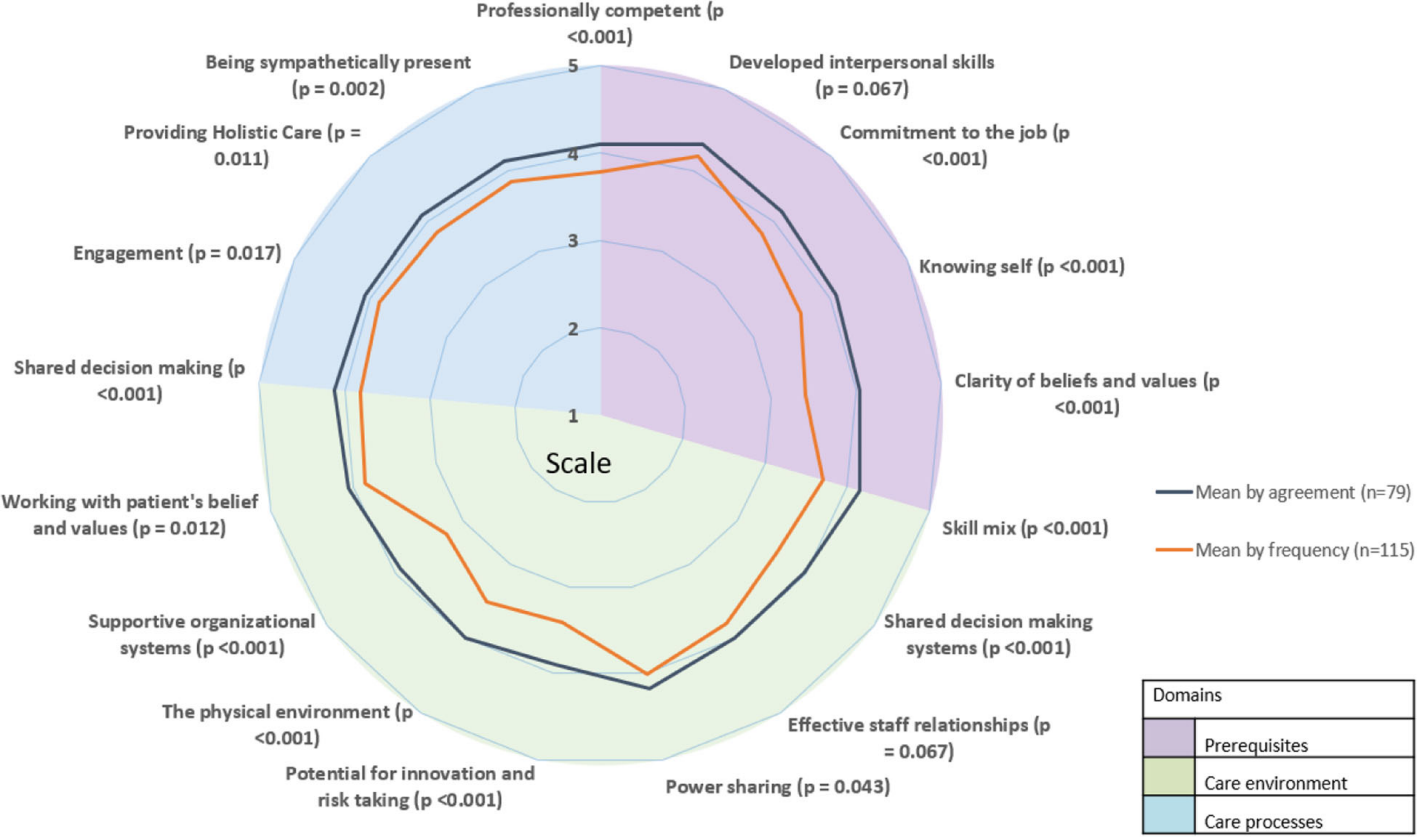
Comparison of mean score between agreement- and frequency-based scales

The scale was then changed to a 5-point frequency rating of “never – rarely – sometimes – often – very often” to adequately capture the level of practice. Subsequent pre-tests indicated differences across constructs, with the lowest mean score in “supportive organizational systems” (mean score 3.2 ± 0.68), “potential for innovation and risk-taking” (mean score 3.4 ± 0.62) and “clarity of beliefs and values” (mean score 3.4 ± 0.57). Independent sample t-test showed significant mean differences and *p*-value, favouring the use of frequency-based scale (Fig. 2).

### Phase II

#### Respondents

A total of 1133 participants from 16 public primary care clinics in a southern state of Malaysia were invited to participate in this phase, with an overall response rate of 81.1% (Table 3). Thirty-five respondents repeated the questionnaire 2 weeks later. Respondents’ mean service years was 8.9 ± 6.7 (range 0.1 to 36 years). Nurses, which included matrons, sisters, staff nurses and community nurses, comprised half of the total respondents (50.2%).

**Table 3:** Total respondents (*n* = 919) characteristics and clinic types

Category	Respondents, n (%)	
	Psychometric evaluation	Test-retest
**Profession**	***n =*** **919**	***n =*** **35**
Family Medicine Specialist	8 (0.9)	1 (2.9)
Medical officer	187 (20.3)	9 (25.7)
Pharmacist	99 (10.8)	4 (11.4)
Nurse	461 (50.2)	11 (31.4)
Medical assistant	71 (7.7)	6 (17.1)
Physiotherapist	8 (0.9)	0
Occupational therapist	7 (0.8)	1 (2.9)
Dietician and Nutritionist and	9 (1.0)	1 (2.9)
Others (lab technician and assistant pharmacist)	7 (0.8)	2 (5.7)
Unknown	62 (6.7)	**–**
**Clinic type (patient attendance/day)**	***n =*** **16**	
Type 1 (> 800)	8	
Type II (500–799)	4	
Type III (300–499)	4	

#### Mean scores

Mean scores ranged from 2.92 to 4.39 and were mostly positively scored. The higher scores coincided with high ceiling effects in 21 items (Table 4).

**Table 4:** Descriptive items and scale characteristics (*n* = 919)

Item	Mean (SD)	Floor effects, n (%)	Ceiling effects, n (%)	Item-scale correlation^**a**^	α	Test-retest ICC (***n*** = 35)
Overall	–	–	–	–	0.96	0.6
Domain 1: Prerequisites						
Professionally Competent	–	–	–	–	0.64	0.82
a1	3.83 (0.73)	6 (0.7)	138 (15.2)	0.46	–	–
a2	3.74 (0.67)	2 (0.2)	86 (9.5)	0.47	–	–
a3	3.95 (0.70)	2 (0.2)	187 (20.5)	0.42	–	–
Developed Interpersonal Skills					0.76	0.78
b4	4.20 (0.58)	–	258 (28.3)	0.53	–	–
b5	4.39 (0.57)	–	392 (42.9)	0.57	–	–
b6	4.07 (0.70)	1 (0.1)	242 (26.4)	0.62	–	–
b7	3.86 (0.69)	1 (0.1)	143 (15.7)	0.54	–	–
Commitment to the job					0.8	0.8
c8	4.24 (0.56)	–	281 (30.8)	0.56	–	–
c9	3.82 (0.73)	2 (0.2)	147 (16.1)	0.6	–	–
c10	3.58 (0.81)	9 (1.0)	97 (10.6)	0.59	–	–
c11	4.10 (0.61)	–	214 (23.4)	0.55	–	–
c12	4.02 (0.64)	2 (0.2)	182 (19.9)	0.61	–	–
Knowing ‘Self’					0.76	0.32
d13	3.81 (0.70)	2 (0.2)	129 (14.1)	0.59	–	–
d14	3.89 (0.68)	1 (0.1)	143 (15.6)	0.66	–	–
d15	3.98 (0.73)	6 (0.7)	192 (21.0)	0.54	–	–
Clarity of Beliefs and Values					0.59	0.76
e16	3.61 (0.80)	13 (1.4)	94 (10.3)	0.4	–	–
e17	2.92 (0.91)	52 (5.7)	36 (3.9)	0.38	–	–
e18	4.04 (0.68)	3 (0.3)	205 (22.4)	0.41	–	–
Domain 2: Care Environment						
Appropriate Skill Mix					0.64	0.59
f19	3.62 (0.75)	8 (0.9)	90 (9.8)	0.47	–	–
f20	3.42 (0.78)	20 (2.2)	47 (5.2)	0.51	–	–
f21	4.16 (0.60)	1 (0.1)	241 (26.3)	0.38	–	–
Shared Decision Making Systems					0.83	0.67
g22	3.94 (0.72)	8 (0.9)	166 (18.1)	0.61	–	–
g23	3.53 (0.95)	39 (4.3)	110 (12.0)	0.73	–	–
g24	3.44 (0.85)	25 (2.7)	71 (7.8)	0.72	–	–
g25	3.45 (0.91)	39 (4.3)	80 (8.8)	0.6	–	–
Effective Staff Relationship					0.75	0.58
h26	3.67 (0.77)	15 (1.6)	88 (9.7)	0.65	–	–
h27	3.80 (0.72)	6 (0.7)	113 (12.4)	0.69	–	–
h28	4.06 (0.63)	–	206 (22.5)	0.43	–	–
Power Sharing					0.75	0.81
i29	4.26 (0.63)	1 (0.1)	325 (35.5)	0.5	–	–
i30	3.87 (0.73)	9 (1.0)	149 (16.3)	0.56	–	–
i31	3.98 (0.75)	4 (0.4)	213 (23.3)	0.59	–	–
i32	3.66 (0.78)	8 (0.9)	97 (10.6)	0.54	–	–
Potential for Innovation and Risk Taking					0.66	0.8
j33	3.73 (0.77)	7 (0.8)	113 (12.4)	0.45	–	–
j34	3.60 (0.85)	25 (2.7)	90 (9.9)	0.49	–	–
j35	3.70 (0.90)	31 (3.4)	131 (14.3)	0.48	–	–
The Physical Environment					0.73	0.67
k36	3.92 (0.71)	6 (0.7)	164 (17.9)	0.56	–	–
k37	3.60 (0.86)	25 (2.7)	98 (10.7)	0.58	–	–
k38	3.55 (0.76)	12 (1.3)	67 (7.3)	0.51	–	–
Supportive Organizational Systems					0.86	0.56
l39	3.60 (0.87)	18 (2.0)	111 (12.2)	0.6	–	–
l40	3.35 (0.96)	41 (4.5)	83 (9.1)	0.7	–	–
l41	3.24 (0.95)	55 (6.0)	49 (5.4)	0.71	–	–
l42	3.49 (0.80)	16 (1.8)	56 (6.1)	0.71	–	–
l43	3.44 (0.85)	21 (2.3)	60 (6.6)	0.65	–	–
Domain 3: Care Processes						
Working with Patient’s Beliefs and Values					0.83	0.71
m44	3.72 (0.70)	5 (0.5)	85 (9.3)	0.59	–	–
m45	3.81 (0.69)	4 (0.4)	105 (11.5)	0.67	–	–
m46	3.70 (0.71)	6 (0.7)	87 (9.5)	0.68	–	–
m47	3.81 (0.68)	1 (0.1)	113 (12.4)	0.7	–	–
Shared Decision Making					0.77	0.76
n48	3.73 (0.78)	10 (1.1)	117 (12.8)	0.63	–	–
n49	3.82 (0.72)	7 (0.8)	119 (13.0)	0.66	–	–
n50	3.91 (0.66)	2 (0.2)	143 (15.7)	0.51	–	–
Engaging authentically					0.8	0.68
o51	3.99 (0.59)	–	150 (16.4)	0.63	–	–
o52	3.80 (0.61)	1 (0.1)	81 (8.9)	0.65	–	–
o53	3.89 (0.61)	1 (0.1)	113 (12.4)	0.64	–	–
Providing Holistic Care					0.77	0.69
p54	3.76 (0.65)	4 (0.4)	75 (8.2)	0.63	–	–
p55	3.77 (0.65)	2 (0.2)	87 (9.5)	0.66	–	–
p56	3.99 (0.57)	–	139 (15.2)	0.55	–	–
Being sympathetically present					0.86	0.73
q57	3.94 (0.63)	1 (0.1)	137 (15.0)	0.68	–	–
q58	3.86 (0.63)	1 (0.1)	111 (12.2)	0.75	–	–
q59	3.91 (0.64)	1 (0.1)	129 (14.2)	0.75	–	–

*SD* standard deviation, *α* Cronbach’s alpha, *ICC* Intraclass Correlation Coefficient, a correlation between item

#### Internal consistency

The overall Cronbach’s alpha for the PCPI-S questionnaire was satisfactory (α = 0.96), indicating that the items measured the same underlying constructs. Several domains had values lower than 0.7, possibly due to the small amount of variance among the respondents and fewer items constituting the construct. The item-scale correlations were generally good, with all item-scale correlations between 0.4 and 0.75.

#### Test-retest reliability

The test–retest reliability of the questionnaire was satisfactory, with an intra-class correlation coefficient of 0.60, (95% CI: 0.49–0.73, *p* < .001). The correlation coefficients for each item by domain are shown in Table 4.

#### Exploratory factor analysis

The Kaiser-Meyer-Olkin (KMO) measure of sampling adequacy of 0.953 and significance of Bartlett’s test of sphericity (χ2 (1711) = 25,404.292, *p* < .005) signified data adequacy for analysis through data reduction procedure. The total variance explained at 60.85% was considered acceptable.

**Fig. 3:**
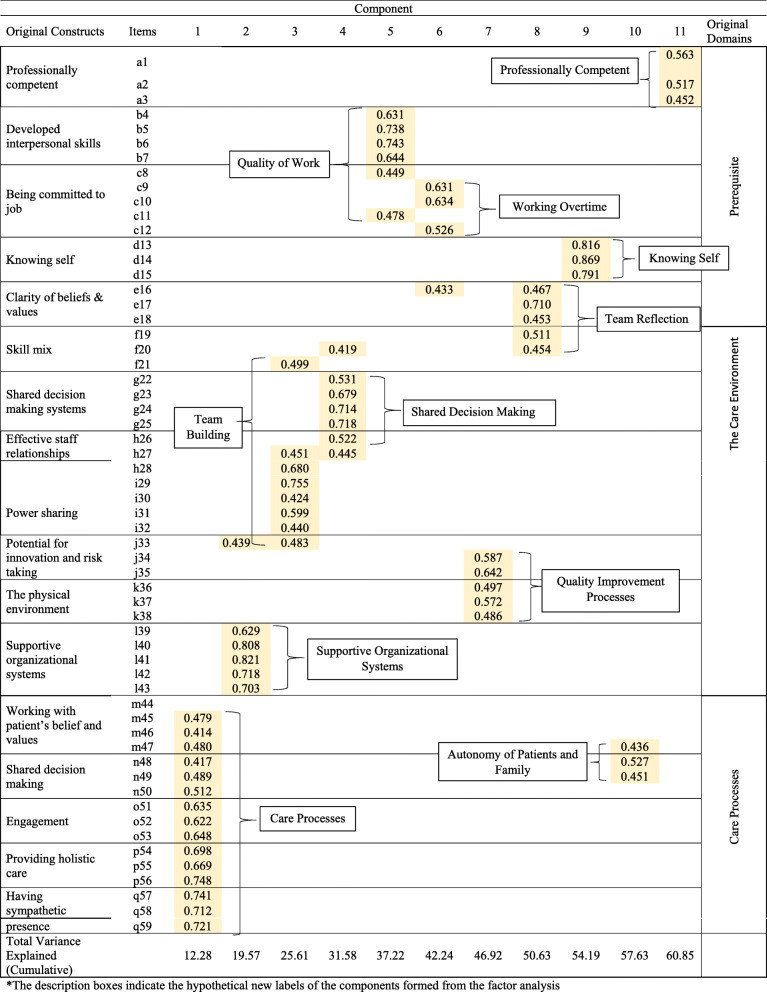
Components and Total Variance Explained

Figure 3 shows the item factor loadings and 11 components that emerged with a computed Eigenvalue greater than 1.

#### Factor interpretation

The 11 components differed from the original 17 constructs that formed the PCPI-S instrument. Component 1 included all the items of domain ‘Care Processes’ of the framework, except for m44 which did not achieve the minimum loading. Components 2, 9 and 11 were noted similar to the original constructs of ‘Supportive Organisational Systems’, ‘Knowing Self’ and ‘Professionally Competent’. Component 3 was deemed related to ‘team building’, as all 8 items pointed towards encouraging one another towards improvement. Component 4 comprised of all the same 5 items construct ‘shared decision-making systems’ with the addition of h26, interpreted as an embedded process of decision-making in the clinic. Component 5 was associated with ‘quality of work’ in which healthcare providers strived to give their best in the assigned tasks within the allocated working hours. Component 6 was closely linked to ‘working overtime’ or needing extra time to perform certain tasks. Cognitive debriefing findings indicated that respondents felt that some tasks (specifically items c9, c10 and c12) were only feasible to be carried out after working hours or on weekends. This was due to the high workload and busy schedule in the public primary care clinics. Component 7 was centred on ‘quality improvement processes’ which included care enhancement and consideration of physical environment. Component 8 was possibly explained by ‘team reflection’ from items that denoted recognition of the need for skill mix within a team. An overarching theme of ‘autonomy in patients and family members’ was considered to suit component 10. However, these 3 items were also closely loaded into component 1, suggesting that the autonomous decision-making may also occur during the patient care process.

## Discussion

The cross-cultural adaptation, translation and psychometric evaluation of the PCPI-S questionnaire were the outcomes of this study. The questionnaire adaptation strongly emphasised on the culture, context, language and local practice for the tool to be used in Malaysia. The challenge as in other studies lies in balancing content validity at conceptual level to allow comparability and being culturally and linguistically accurate [11, 26–28]. In this context, a term or phrase in the original questionnaire used in a different country may vary significantly when used in a different language [26]. Among the few items observed in this study, the translated terms used were also heavily influenced by culture, rendering the translation conceptually different. These problems encountered were similar to the Norwegian study [8]. The pre-testing process in this study was therefore optimized to achieve conceptual, semantic, idiomatic, and experiential equivalence between the original and translated questionnaires [29]. The interpretation of the behaviour codes was also vigilantly reviewed by item. Higher number of codes suggested more respondents had comprehension problems but not necessarily reflecting the magnitude associated with the item.

Rescaling the 5-point Likert scale to frequency-based was justified to capture their level of person-centred practice in the clinics more meaningfully. This also avoided presence of acquiescence bias whereby respondents had the propensity to agree in the original agreement-based scale [30]. As observed in earlier pre-tests in this study, respondents replied in a way that corresponds with what is perceived as “desirable” personality characteristics. The choice of the scale’s evaluative dimension has therefore an impact on data quality.

This study showed similar mean scores to the other PCPI-S studies [7, 8]. The high ceiling effects may be explained with two possibilities. Firstly, this may imply a limited content validity and responsiveness of the instrument [19]. Alternatively, the findings could be modestly accurate in representing person-centred practice in Malaysia, ensued from the Health Ministry’s vision and mission on providing a customer-centred care [31].

The components established from factor exploration differed from the original questionnaire. While some original components were retained, others collapsed into a broader new group. For instance, almost all items from the domain ‘Care Processes’ formed a single component. The overarching theme of care processes indicated meaningful interactions between service providers and users (patients and their families) in activities that constitute healthcare - including diagnosis, treatment, rehabilitation, prevention and patient education. New components were formed from a combination of items originating from different constructs. Our exploration found differing local context and culture influenced the healthcare providers’ practice, hence influencing factor interpretation. The overall conceptual ideas of person-centred practice remained similar.

One of the major strengths of this study is the extensive exploration of culture, context and language in influencing the cross-cultural adaptation process, which not only ensured translation accuracy but validity of the instrument. Findings allowed greater understanding on what constitutes local person-centred practice and how such practice comes into place. By enhancing its contextualization and practicality, clinics will be inclined to utilise the questionnaire findings for their practice. We believe that our contribution in the development of this locally validated tool could allow researchers and stakeholders to assess person-centred practices in the Asian region. To our knowledge, this was the first study that investigated the reliability and validity of the PCPI-S questionnaire in a primary care context, which supports and extends applicability of the instrument in various healthcare settings.

However, this study has certain limitations. The cognitive debriefing process by itself can indicate the existence of problems with questions but not the quantitative information on the quality of self-reported data. To obtain this type of information, studies comparing self-reported data to data from alternative sources are required. Secondly, this study was only tested among public primary healthcare providers. The conceptual, cultural, linguistic influence, as well as formation of the components seen in this study might be heavily influenced by healthcare practice, and therefore might render different results if tested in other fields or backgrounds. While the constructs should be viewed with caution, the findings may be useful for comparison in follow-up assessment of the underlying structure.

## Conclusion

The adapted PCPI-S questionnaire can be a practicable instrument in assessing the Malaysian person-centred practice among primary healthcare providers. The influence of culture, context, language as well as local practice should be taken into consideration and tested again should this instrument be used in a different set of population in the future, or when translating evidence into person-centred practice. Altogether, the present study makes an important contribution to the reliability and underlying structure of person-centred practice in a primary care background.

## Supplementary Information

**Additional file 1.** Cognitive debriefing probing questions.

**Additional file 2.** Summary of debriefing codes according to items in each pre-test sessions.

**Additional file 3.** Summary of items modifications and final translated version.

## Data Availability

The dataset that supports the findings of this article belongs to the Primary Care Systems for Person-Centred Provider Practices study. Requests for the data can be obtained from Dr. Mohd Azahadi Omar (drazahadi@moh.gov.my), the head of sector for Biostatistics & Data Repository, National Institute of Health, Ministry of Health Malaysia and with the permission from the Director-General of Health, Malaysia.

## Abbreviations

PCC: Person-Centred Care
EFA: Exploratory Factor Analysis
KMO: Kaiser-Meyer-Olkin
PCPI-S: Person-centred Practice Inventory-Staff
