# Supplementary material for: Cross-cultural adaptation and exploratory factor analysis of the Person-centred Practice Inventory - Staff (PCPI-S) questionnaire among Malaysian primary healthcare providers
Source: BMC Health Serv Res. 2021 Jan 7;21:32. doi: 10.1186/s12913-020-06012-9 (PMC7792065; doi:10.1186/s12913-020-06012-9)
Supplement: Supplementary file 1 — Additional file 1. Cognitive debriefing probing questions. [file 12913_2020_6012_MOESM1_ESM.docx]

**S1 Appendix: Cognitive debriefing probing questions**

| **No** | **Probing questions** |
| --- | --- |
| Q1 | Apa yang anda faham dengan **‘jagaan kesihatan’**?  Apa yang anda faham dengan **‘membincangkan’** dalam ayat ini? Sekiranya diganti dengan perkataan **‘merundingkan’**, adakah maksudnya berubah? Bagaimana? |
| Q2 | Apa yang anda faham dengan **‘memberikan perhatian melebihi daripada tugasan hakiki sedia ada’?** |
| Q3 | Apa yang anda faham dengan **‘kompetensi professional’?** |
| Q7 | Apa yang anda faham dengan **‘penglibatan dan kerjasama’?** |
| Q8 | Adakah perbezaan antara ‘**berusaha**’ dengan ‘**berusaha dengan gigih’?** |
| Q10 | Apa yang anda faham dengan ‘**berusaha melebihi kebiasaan saya’**? Adakah maksudnya sama dengan berusaha sedaya upaya? |
| Q11 | Adakah perbezaan antara ‘**berkualiti’** dengan ‘**berkualiti tinggi’?** |
| Q12 | Soalan ini merujuk kepada pengalaman jagaan siapa - diri sendiri atau pesakit? |
| Q14 | Adakah anda memahami maksud ‘**bermuhasabah**’? Jika ya, apakah maksud yang anda fahami? |
| Q17 | Apa yang anda faham dengan **‘mempertikai’** di sini? |
| Q20 | What do you understand by **‘I am able to make the case’**? |
| Q22 | What do you understand by **‘to inform my decision making’**? Adakah ia sama maksud dengan ‘**memaklumkan keputusan saya’?** |
| Q23 | What do you understand by ‘**forum wide decision making’**? Adakah ia sama maksud dengan **‘perbincangan bagi membuat keputusan dalam organisas**i’? Organisasi dalam soalan ini merujuk kepada peringkat pentadbiran yang mana? |
| Q25 | Apa yang anda faham dengan ‘**pendapat saya diminta dalam perbincangan’**? |
| Q29 | Apa yang anda faham dengan **‘dikenalpasti’**?  Apakah yang dimaksudkan dengan ‘**diiktiraf’** dalam konteks kerja anda? |
| Q30 | **‘Matlamat yang dikongsi bersama’** merujuk kepada matlamat siapa? |
| Q31 | Apa yang anda faham dengan **‘memudahcara’?** |
| Q32 | Apa yang anda faham dengan ‘**pembangunan amalan kerja’?** |
| Q34 | Apa yang anda faham dimaksudkan dalam soalan ini?  Apa yang anda faham dengan ‘**pengambilan risiko’**? |
| Q41 | Apa yang anda faham dengan **‘dikenali’** dalam soalan ini? |
| Q42 | Apa yang dimaksudkan dengan **‘menyuarakan kebimbangan’** dalam soalan ini? |
| Q50 | **‘Anggota kesihatan yang lain’** merujuk kepada siapa? |
