# Supplementary material for: Cross-cultural adaptation and exploratory factor analysis of the Person-centred Practice Inventory - Staff (PCPI-S) questionnaire among Malaysian primary healthcare providers
Source: BMC Health Serv Res. 2021 Jan 7;21:32. doi: 10.1186/s12913-020-06012-9 (PMC7792065; doi:10.1186/s12913-020-06012-9)
Supplement: Supplementary file 2 — Additional file 2. Summary of debriefing codes according to items in each pre-test sessions. [file 12913_2020_6012_MOESM2_ESM.docx]

**S2 Appendix: Summary of debriefing codes according to items in each pre-test sessions**

| **Item** | **Pre-test round** | **Codes** | | | | | |
| --- | --- | --- | --- | --- | --- | --- | --- |
|  |  | **1: Request for clarification** | **2: Answer with uncertainty, misunderstanding** | **3: Disagree with terms/sentences used** | **4: Don’t know/wrong interpretation** | **5: Not applicable/Non response** | **6: Translated version carries different meaning** |
| **A1** | 1 | 1 |  | 2 | 6 |  | 2 |
|  | 2 |  | 1 | 2 | 7 |  | 2 |
|  | 3 |  | 1 |  | 3 | 2 |  |
|  | 4 | 1 |  |  |  |  |  |
|  | 5 |  | 1 | 1 | 3 | 1 |  |
| **A2** | 1 | 2 |  | 1 |  |  |  |
|  | 2 | 4 |  |  | 3 | 1 | 2 |
|  | 3 | 2 | 2 |  |  |  | 1 |
|  | 4 |  |  |  |  |  |  |
|  | 5 | 1 |  |  |  |  |  |
| **A3** | 1 | 1 |  |  | 1 |  |  |
|  | 2 |  |  |  |  |  |  |
|  | 3 |  |  |  |  |  |  |
|  | 4 |  |  |  | 1 |  |  |
|  | 5 | 1 |  |  |  |  |  |
| **B4** | 1 | 4 |  |  |  |  |  |
|  | 2 |  |  |  |  |  |  |
|  | 3 |  |  |  |  |  |  |
|  | 4 |  | 1 |  |  |  |  |
|  | 5 |  |  |  |  |  |  |
| **B5** | 1 |  |  |  |  |  |  |
|  | 2 |  |  |  |  |  |  |
|  | 3 |  |  |  |  |  |  |
|  | 4 |  |  |  |  |  |  |
|  | 5 |  |  |  |  |  |  |
| **B6** | 1 |  |  |  |  |  | 1 |
|  | 2 | 1 |  |  |  |  |  |
|  | 3 | 1 |  |  |  |  |  |
|  | 4 |  |  |  |  |  |  |
|  | 5 |  |  |  |  |  |  |
| **B7** | 1 | 2 | 1 | 4 |  |  |  |
|  | 2 | 2 |  |  | 1 |  |  |
|  | 3 |  |  |  |  |  |  |
|  | 4 |  |  |  |  |  |  |
|  | 5 | 1 | 1 |  |  |  |  |
| **C8** | 1 |  |  | 1 |  |  |  |
|  | 2 | 1 |  |  | 4 |  |  |
|  | 3 |  |  |  |  |  |  |
|  | 4 |  |  |  |  |  |  |
|  | 5 |  |  |  |  |  |  |
| **C9** | 1 |  |  |  |  |  |  |
|  | 2 |  |  |  |  |  |  |
|  | 3 | 1 |  |  |  |  |  |
|  | 4 |  |  |  |  |  |  |
|  | 5 |  |  |  |  |  |  |
| **C10** | 1 | 4 | 1 | 3 | 2 |  | 1 |
|  | 2 | 2 |  | 3 |  |  | 1 |
|  | 3 |  |  |  | 1 |  |  |
|  | 4 |  |  |  |  |  |  |
|  | 5 | 2 |  |  |  |  |  |
| **C11** | 1 |  |  |  |  |  |  |
|  | 2 |  |  |  |  |  |  |
|  | 3 |  |  |  |  |  |  |
|  | 4 |  |  |  |  |  |  |
|  | 5 |  |  |  |  |  |  |
| **C12** | 1 | 10 | 1 | 1 | 1 |  | 2 |
|  | 2 | 2 |  |  |  |  | 1 |
|  | 3 | 1 | 2 |  |  |  |  |
|  | 4 | 1 |  |  |  |  |  |
|  | 5 |  |  |  |  |  |  |
| **D13** | 1 | 1 | 2 | 1 | 2 |  |  |
|  | 2 | 4 | 1 | 1 | 2 |  | 1 |
|  | 3 |  |  |  |  |  |  |
|  | 4 |  |  |  |  |  |  |
|  | 5 |  |  |  |  |  |  |
| **D14** | 1 | 1 |  |  | 3 |  |  |
|  | 2 | 2 | 4 | 3 | 6 |  | 2 |
|  | 3 | 1 |  |  | 3 |  | 2 |
|  | 4 |  |  |  |  |  |  |
|  | 5 |  |  | 1 |  |  |  |
| **D15** | 1 | 1 |  |  |  |  |  |
|  | 2 |  |  |  | 5 |  |  |
|  | 3 |  |  |  | 1 |  |  |
|  | 4 |  | 2 |  |  |  |  |
|  | 5 |  |  |  |  |  |  |
| **E16** | 1 |  |  |  |  |  |  |
|  | 2 |  |  |  |  |  |  |
|  | 3 |  |  |  |  |  |  |
|  | 4 |  |  |  |  |  |  |
|  | 5 | 1 |  |  |  |  |  |
| **E17** | 1 | 3 |  | 7 |  |  |  |
|  | 2 | 3 |  | 9 |  | 1 |  |
|  | 3 |  |  |  |  |  |  |
|  | 4 |  |  |  |  |  |  |
|  | 5 |  |  |  |  |  |  |
| **E18** | 1 |  |  | 3 |  |  | 1 |
|  | 2 |  |  |  |  |  |  |
|  | 3 |  |  |  |  |  |  |
|  | 4 |  |  |  |  |  |  |
|  | 5 |  |  |  |  |  |  |
| **F19** | 1 |  |  |  |  |  |  |
|  | 2 |  |  |  | 1 | 1 |  |
|  | 3 |  |  |  |  |  |  |
|  | 4 |  |  |  |  |  |  |
|  | 5 |  |  |  |  |  |  |
| **F20** | 1 | 4 | 1 | 1 |  |  |  |
|  | 2 | 3 | 1 | 1 | 2 | 1 | 1 |
|  | 3 |  | 5 |  | 1 |  | 1 |
|  | 4 | 1 |  |  |  |  |  |
|  | 5 |  |  |  | 1 | 1 |  |
| **F21** | 1 |  |  |  |  |  |  |
|  | 2 |  |  |  |  |  |  |
|  | 3 | 1 |  |  |  |  |  |
|  | 4 |  |  |  |  |  |  |
|  | 5 |  |  |  |  |  |  |
| **G22** | 1 | 2 |  | 1 |  |  |  |
|  | 2 |  | 1 |  | 6 |  | 1 |
|  | 3 |  |  |  |  |  |  |
|  | 4 |  |  |  |  |  |  |
|  | 5 |  |  |  | 1 |  |  |
| **G23** | 1 |  | 1 |  |  |  |  |
|  | 2 |  |  |  |  |  |  |
|  | 3 |  |  |  |  |  |  |
|  | 4 | 1 |  |  |  |  |  |
|  | 5 |  |  |  |  |  |  |
| **G24** | 1 |  |  | 1 |  |  |  |
|  | 2 |  |  |  |  |  |  |
|  | 3 |  |  |  |  |  |  |
|  | 4 |  |  |  |  |  |  |
|  | 5 |  |  |  |  |  |  |
| **G25** | 1 |  |  |  |  |  |  |
|  | 2 |  |  |  |  |  |  |
|  | 3 |  |  |  |  |  |  |
|  | 4 |  |  |  |  |  |  |
|  | 5 |  |  |  |  | 1 |  |
| **H26** | 1 |  |  |  |  |  |  |
|  | 2 | 3 |  | 1 | 2 |  |  |
|  | 3 |  |  |  |  |  |  |
|  | 4 | 3 |  |  |  |  |  |
|  | 5 | 1 | 1 |  |  |  |  |
| **H27** | 1 | 2 |  | 1 |  |  |  |
|  | 2 | 1 |  |  | 1 |  |  |
|  | 3 |  |  |  |  |  |  |
|  | 4 | 3 |  |  |  |  |  |
|  | 5 |  |  |  |  |  |  |
| **H28** | 1 | 2 |  |  | 1 |  |  |
|  | 2 | 3 |  |  | 1 |  |  |
|  | 3 | 1 |  |  |  |  |  |
|  | 4 |  |  |  |  |  |  |
|  | 5 |  |  |  |  |  |  |
| **I29** | 1 | 2 |  |  |  |  | 1 |
|  | 2 |  |  |  |  |  |  |
|  | 3 |  |  |  |  |  | 2 |
|  | 4 |  |  |  |  |  |  |
|  | 5 |  |  |  |  |  |  |
| **I30** | 1 |  |  |  |  |  |  |
|  | 2 | 1 |  |  |  |  |  |
|  | 3 | 2 |  |  |  |  |  |
|  | 4 |  |  |  |  |  |  |
|  | 5 |  |  |  |  |  |  |
| **I31** | 1 | 2 |  | 4 |  |  |  |
|  | 2 |  |  |  |  |  |  |
|  | 3 |  |  |  |  |  |  |
|  | 4 | 1 |  |  |  |  |  |
|  | 5 |  |  |  |  |  |  |
| **I32** | 1 |  |  |  |  |  | 1 |
|  | 2 | 4 |  | 1 |  |  |  |
|  | 3 | 1 |  |  |  |  |  |
|  | 4 |  |  |  |  |  |  |
|  | 5 |  |  |  | 1 |  |  |
| **J33** | 1 | 1 |  | 1 |  |  |  |
|  | 2 | 2 |  | 1 | 5 |  |  |
|  | 3 |  |  |  |  |  |  |
|  | 4 |  |  |  |  |  |  |
|  | 5 |  |  |  |  |  |  |
| **J34** | 1 | 3 |  |  | 3 |  |  |
|  | 2 | 2 | 1 | 1 | 10 | 1 |  |
|  | 3 |  | 1 |  |  |  |  |
|  | 4 |  |  |  |  |  |  |
|  | 5 |  |  |  |  |  |  |
| **J35** | 1 | 2 | 1 | 2 |  |  | 1 |
|  | 2 | 3 |  | 2 | 1 | 1 |  |
|  | 3 | 3 |  |  | 1 |  | 1 |
|  | 4 |  |  |  |  |  |  |
|  | 5 |  |  |  |  |  | 1 |
| **K36** | 1 | 1 |  |  |  |  |  |
|  | 2 |  |  |  |  |  |  |
|  | 3 |  |  |  |  |  |  |
|  | 4 |  |  | 1 |  |  |  |
|  | 5 |  |  |  |  |  |  |
| **K37** | 1 | 2 | 1 | 4 |  |  |  |
|  | 2 | 5 |  | 7 | 6 |  |  |
|  | 3 | 2 |  | 1 | 1 |  | 5 |
|  | 4 |  | 1 |  |  |  |  |
|  | 5 |  |  |  |  |  |  |
| **K38** | 1 |  |  |  |  |  |  |
|  | 2 |  |  | 1 |  |  |  |
|  | 3 |  |  |  |  |  |  |
|  | 4 | 1 |  |  |  |  |  |
|  | 5 |  |  |  |  |  |  |
| **L39** | 1 |  |  |  |  |  |  |
|  | 2 |  |  |  |  |  |  |
|  | 3 | 1 |  |  |  |  |  |
|  | 4 |  |  |  |  |  |  |
|  | 5 |  |  |  |  |  |  |
| **L40** | 1 | 1 |  |  |  |  |  |
|  | 2 |  |  |  |  |  |  |
|  | 3 |  |  |  |  |  |  |
|  | 4 |  |  |  |  |  |  |
|  | 5 |  |  |  |  |  |  |
| **L41** | 1 | 1 |  | 1 |  |  |  |
|  | 2 |  |  | 6 |  |  |  |
|  | 3 |  |  |  |  |  |  |
|  | 4 |  |  |  |  |  |  |
|  | 5 |  |  |  |  |  |  |
| **L42** | 1 |  |  | 2 |  |  |  |
|  | 2 | 1 |  |  |  |  |  |
|  | 3 |  |  |  |  |  |  |
|  | 4 |  |  |  |  |  |  |
|  | 5 |  |  |  | 1 |  |  |
| **L43** | 1 |  |  |  |  |  |  |
|  | 2 |  |  |  |  |  |  |
|  | 3 |  |  |  |  |  |  |
|  | 4 |  |  |  |  |  |  |
|  | 5 |  |  |  |  |  |  |
| **M44** | 1 | 2 |  | 3 | 2 | 2 | 1 |
|  | 2 | 1 |  | 1 | 7 |  | 1 |
|  | 3 |  | 1 |  | 2 |  |  |
|  | 4 | 1 |  |  | 3 |  |  |
|  | 5 | 1 |  |  |  |  |  |
| **M45** | 1 |  |  |  |  |  |  |
|  | 2 |  |  |  |  |  |  |
|  | 3 |  |  |  |  |  |  |
|  | 4 |  | 1 |  |  |  |  |
|  | 5 |  |  |  |  |  |  |
| **M46** | 1 | 1 |  |  | 2 |  |  |
|  | 2 |  |  | 1 | 4 |  | 1 |
|  | 3 |  |  |  |  |  | 1 |
|  | 4 |  |  | 3 |  |  |  |
|  | 5 |  |  |  | 1 |  |  |
| **M47** | 1 |  |  |  |  |  |  |
|  | 2 | 1 | 1 |  |  |  |  |
|  | 3 |  |  |  |  |  |  |
|  | 4 |  |  |  |  |  |  |
|  | 5 |  |  |  |  |  |  |
| **N48** | 1 | 1 |  |  |  |  |  |
|  | 2 |  |  |  |  |  | 1 |
|  | 3 |  |  |  |  |  |  |
|  | 4 | 10 |  |  |  |  |  |
|  | 5 |  |  |  |  |  |  |
| **N49** | 1 |  |  | 1 |  |  |  |
|  | 2 | 1 |  |  |  |  |  |
|  | 3 |  |  |  |  |  |  |
|  | 4 |  |  | 1 | 1 |  |  |
|  | 5 |  |  |  |  |  |  |
| **N50** | 1 | 1 | 1 | 3 |  |  |  |
|  | 2 |  |  | 1 |  |  |  |
|  | 3 |  |  |  |  |  |  |
|  | 4 |  |  | 4 |  |  |  |
|  | 5 |  |  |  |  |  |  |
| **O51** | 1 |  |  | 1 |  |  |  |
|  | 2 | 2 |  |  |  |  |  |
|  | 3 |  |  |  |  |  |  |
|  | 4 |  |  |  |  |  |  |
|  | 5 |  |  |  |  |  |  |
| **O52** | 1 |  |  | 1 |  |  |  |
|  | 2 | 1 |  |  |  |  |  |
|  | 3 |  | 1 |  | 1 |  |  |
|  | 4 | 2 |  |  |  |  |  |
|  | 5 |  |  |  |  |  |  |
| **O53** | 1 | 1 |  | 1 |  |  |  |
|  | 2 |  |  |  |  |  |  |
|  | 3 |  | 1 |  | 3 |  |  |
|  | 4 |  |  |  |  |  |  |
|  | 5 |  |  |  |  |  |  |
| **P54** | 1 | 1 |  | 1 |  |  |  |
|  | 2 | 2 |  |  |  |  |  |
|  | 3 |  |  |  |  |  |  |
|  | 4 |  |  |  |  |  |  |
|  | 5 |  |  |  |  |  |  |
| **P55** | 1 |  |  |  |  |  |  |
|  | 2 | 2 |  |  |  |  |  |
|  | 3 |  |  |  |  |  |  |
|  | 4 |  |  |  |  |  |  |
|  | 5 |  |  |  |  |  |  |
| **P56** | 1 |  |  | 1 |  |  |  |
|  | 2 |  |  |  |  |  |  |
|  | 3 |  |  |  |  |  |  |
|  | 4 |  |  |  |  |  |  |
|  | 5 |  |  |  |  |  |  |
| **Q57** | 1 |  |  |  |  |  |  |
|  | 2 | 1 |  |  |  |  |  |
|  | 3 |  |  |  |  |  |  |
|  | 4 |  |  |  |  |  |  |
|  | 5 |  |  |  |  |  |  |
| **Q58** | 1 |  |  |  |  |  |  |
|  | 2 |  |  |  |  |  |  |
|  | 3 |  |  |  |  |  |  |
|  | 4 |  | 6 |  | 1 |  |  |
|  | 5 |  |  |  |  |  |  |
| **Q59** | 1 |  |  |  |  |  |  |
|  | 2 |  |  |  |  |  |  |
|  | 3 |  |  |  |  |  |  |
|  | 4 |  |  | 2 |  | 2 |  |
|  | 5 |  |  |  |  |  |  |
