# Supplementary material for: Cross-cultural adaptation and exploratory factor analysis of the Person-centred Practice Inventory - Staff (PCPI-S) questionnaire among Malaysian primary healthcare providers
Source: BMC Health Serv Res. 2021 Jan 7;21:32. doi: 10.1186/s12913-020-06012-9 (PMC7792065; doi:10.1186/s12913-020-06012-9)
Supplement: Supplementary file 3 — Additional file 3. Summary of items modifications and final translated version. [file 12913_2020_6012_MOESM3_ESM.docx]

**S3 Appendix: Summary of items modifications and final translated version**

| **Item** | **Original** | **Issues** | **Findings and Modifications** | **Final Malay Translation** |
| --- | --- | --- | --- | --- |
| A1 | I have the necessary skills to negotiate care options | Complex sentence with grammatical and multiple meanings | PT*1: Item lengthy and difficult to understand  PT 2: Sentence simplified  PT 3: The term *‘pelbagai’* (varieties) was added but respondents still had difficulty understanding the item  PT4: Example added | *Saya mahir membincangkan pelbagai pilihan jagaan kesihatan.*  *(cth: Jururawat berkemahiran membincangkan kaedah-kaedah perancang keluarga)* |
| A2 | When I provide care I pay attention to more than the immediate physical task | Unfamiliarity with subject experience | PT2: ‘Physical task’ was translated to *‘tugas hakiki’* in Malay but was not used in local context. The word *‘tugasan*’ (task) alone was adequate  PT3: Sentence structure modified  PT4: The translated term for ‘immediate’ (*pada waktu itu*) dropped as it served no additional purpose and made the sentence lengthy | *Perhatian yang saya berikan semasa memberi jagaan adalah melebihi dari apa yang diperlukan.* |
| A3 | I actively seek opportunities to extend my professional competence | - | No Modification | *Saya mencari peluang untuk meluaskan kompetensi profesional saya..* |
| B4 | I ensure I hear and acknowledge others perspectives | Ambiguous meaning | PT1: Respondents were confused on whose ‘other perspectives’ the question was referring to  PT2: Target group was added in bracket at the end of the item | *Saya pastikan saya mendengar dan mengambil maklum pandangan orang lain (cth: pesakit, keluarga, rakan sekerja).* |
| B5 | In my communication I demonstrate respect for others | - | No Modification | *Saya menunjukkan rasa hormat apabila berkomunikasi dengan orang lain.* |
| B6 | I use different communication techniques to find mutually agreed solutions | - | No Modification | *Saya menggunakan teknik komunikasi yang berbeza-beza untuk mencapai penyelesaian yang dipersetujui bersama.* |
| B7 | I pay attention to how my non-verbal cues impact on my engagement with others | Similar and compound words | PT2: The term ‘engagement’ was originally translated to ‘*penglibatan dan kerjasama saya*’ (my involvement and cooperation), but these terms were dropped and replaced with ‘*semasa berurusan*’ (when dealing) which adequately represents components of ‘engagement’ | *Saya memberi perhatian bagaimana isyarat bukan lisan saya memberi impak semasa berurusan dengan orang lain.* |
| C8 | I strive to deliver high quality care to people | Words bearing emotional weight and action words | PT1: The term ‘strive’ was translated to a Malay word with similar meaning (‘*berusaha’*). However, respondents associated the term with ‘putting effort’, rather than to make greater or extra effort.  PT2: The Malay word *‘dengan gigih’* (greater effort) was added to the sentence | *Saya berusaha dengan gigih untuk memberi jagaan berkualiti tinggi kepada setiap individu.* |
| C9 | I seek opportunities to get to know people and their families in order to provide holistic care | - | No Modification | *Saya mencari peluang untuk mengenali individu dan keluarga mereka agar dapat memberi jagaan yang menyeluruh.* |
| C10 | I go out of my way to spend time with people receiving care | Words bearing emotional weight and action words | PT 2: Sentence simplified | *Saya berusaha lebih daripada biasa untuk meluangkan masa dengan individu yang menerima rawatan.* |
| C11 | I strive to deliver high quality care that is informed by evidence | - | No Modification | *Saya berusaha gigih memberi jagaan berkualiti tinggi berdasarkan bukti (garis panduan, CPG, SOP, kajian saintifik).* |
| C12 | I continuously look for opportunities to improve the care experiences | Ambiguous meaning | PT1: Respondents were confused on whose ‘care experience’’ the question was referring to  PT2: The term *‘individu*’ (individual) changed to ‘’*semua*’ (all (patient, family, healthcare providers, etc))’ to represent larger target groups | *Saya mencari peluang menambahbaik apa yang dilalui oleh semua (pesakit, keluarga, anggota kesihatan, dsb) dalam jagaan kesihatan.* |
| D13 | I take my time to explore why I react as I do in certain situations | Multiple meanings | PT1: Lengthy sentence  PT2: The translated terms *‘menilai semula’* were misinterpreted to be a negative action, i.e reflection of outcome from poor planning and judgement  PT3: The translated term changed to *‘muhasabah*’ (positive self-reflection). | *Saya mengambil masa bermuhasabah mengapa saya bertindak sebegitu dalam situasi tertentu.* |
| D14 | I use reflection to check out if my actions are consistent with my ways of being | Complex sentence with multiple meanings | PT1: Misinterpreted, difficult to understand  PT2: Sentence shortened, simplified but still difficult to understand  PT3: Specific example added, but skewed respondents' answers  PT4: Example modified to be more general | *Saya menilai semula samada tindakan saya selaras dengan prinsip hidup saya.*  *(cth: Apabila saya melanggar prinsip hidup saya semasa bekerja, saya menilai semula tindakan saya)* |
| D15 | I pay attention to how my life experiences influence my practice | - | No Modification | *Saya memberi perhatian bagaimana pengalaman hidup saya mempengaruhi amalan kerja saya*. |
| E16 | I actively seek feedback from others about my practice | - | No Modification | *Saya berusaha untuk mendapatkan maklumbalas orang lain mengenai amalan kerja saya.* |
| E17 | I challenge colleagues when their practice is inconsistent with our team’s shared values and beliefs | Words bearing emotional weight and action words | No modification across 5 pretests. Main problem with the word *‘mempertikai’* (to challenge). Several translated term options were interpreted as suggesting expression of sharp disapproval or criticism of someone. While alternative translated terms such as ‘negotiate’ or ‘offer’ were suggested, these were not accepted in the finalised version of the questionnaire to carry the intended meaning, which was “to stimulate or trigger by the way of disputing someone”. The term *‘mempertikai’* (to challenge) was retained. | *Saya mempertikai rakan sekerja apabila amalan kerja mereka tidak konsisten dengan nilai dan kepercayaan pasukan.* |
| E18 | I support colleagues to develop their practice to reflect the team’s shared values and beliefs | - | No Modification | *Saya menyokong rakan sekerja mempertingkatkan amalan kerja mereka bagi mencerminkan nilai dan kepercayaan pasukan* |
| F19 | I recognize when there is a deficit in knowledge and skills in the team and its impact on care delivery | - | No Modification | *Saya boleh mengenalpasti wujudnya kekurangan pengetahuan dan kemahiran dalam pasukan, serta kesannya terhadap jagaan kesihatan.* |
| F20 | I am able to make the case when skill mix falls below acceptable levels | No similar word and unfamiliarity with subject experience | PT 1 & 2: Difficult to understand meaning of 'skill mix’ as the term is not commonly used in local context. The definition was however available under construct definition column  PT3: The instruction to refer to construct definition was added | *Saya dapat mengesyorkan sesuatu apabila skill mix* berada di bawah tahap yang boleh diterima*.  **Sila rujuk konstruk untuk definisi skill mix* |
| F21 | I value the input from all team members and their contributions to care | - | No Modification | *Saya menghargai input daripada semua ahli pasukan dan sumbangan mereka kepada jagaan.* |
| G22 | I actively participate in team meetings to inform my decision-making | Grammatical meaning and sentence structuring | PT 1: Misinterpreted  PT2: Translated term modified, but misinterpretation persisted  PT3: Sentence structure changed. Issue resolved. | *Saya mengambil bahagian dalam perbincangan pasukan untuk membantu saya membuat keputusan* |
| G23 | I participate in organization-wide decision making forums that impact on practice | - | No Modification | *Saya mengambil bahagian dalam perbincangan bagi membuat keputusan organisasi (di peringkat fasiliti/ daerah/ negeri/ kebangsaan) yang memberi kesan kepada amalan kerja.* |
| G24 | I am able to access opportunities to actively participate in influencing decisions in my directorate/division | - | No Modification | *Saya berpeluang untuk mengambil bahagian secara aktif dalam mempengaruhi keputusan unit/bahagian saya.* |
| G25 | My opinion is sought in clinical decision-making forums (e.g. ward rounds, case conferences, discharge planning) | - | No Modification | *Pendapat saya diminta dalam perbincangan yang melibatkan keputusan klinikal (cth: perancangan rawatan, sesi kaunseling, lawatan ke rumah).* |
| H26 | I work in a team that values my contribution to person-centred care | Sentence structure | PT1: Sentence too long  PT2: Sentence simplified | *Pasukan saya menghargai sumbangan saya terhadap person-centred care.* |
| H27 | I work in a team that encourages everyone’s contribution to person-centred care | Sentence structure | PT1: Sentence too long  PT2: Sentence simplified | *Pasukan saya menggalakkan sumbangan semua terhadap person-centred care.* |
| H28 | My colleagues positively role model the development of effective relationship | - | No Modification | *Rakan sekerja saya menunjukkan teladan baik dalam membentuk hubungan yang berkesan.* |
| I29 | The contribution of colleagues is recognized and acknowledged | Compound words with multiple meanings | PT1,2: The term ‘recognised and acknowledged’ were translated to two separate terms with the same meaning in Malay *(‘dikenalpasti dan dihargai’).* However, the Malay term *‘dihargai’* alone represented both meanings.  PT3: Single term was used | *Sumbangan rakan sekerja adalah dihargai.* |
| I30 | I actively contribute to the development of shared goals | - | No Modification | *Saya menyumbang kepada pembangunan matlamat bersama.* |
| I31 | The leader facilitates participation | Unfamiliarity with subject experience | PT1: The term *‘memudahcara*’ used to translate ‘facilitates’ was not familiar among many respondents  PT2: Term changed | *Ketua memudah dan melancarkan penglibatan ahli pasukan.* |
| I32 | I am encouraged and supported to lead developments in practices | Sentence structure | PT1: Sentence too long  PT2: Sentence simplified  PT4: The term ‘to lead’ highlighted in bold to help redirect the focus | *Saya diberi galakan dan sokongan untuk* ***memimpin*** *kemajuan amalan kerja* |
| J33 | I am supported to do things differently to improve my practice | Sentence structure | PT1,2: Sentence too long  PT3: Sentence simplified | *Saya disokong untuk melakukan sebarang perubahan bagi meningkatkan amalan kerja saya.* |
| J34 | I am able to balance the use of evidence with taking risks | Complex sentence with ambiguous meaning | PT1: Difficult to understand, misinterpreted  PT2: The term ‘bila perlu’ (when necessary) added but still difficult for respondents to understand question  PT3: Example added | *Saya dapat mengimbangi antara penggunaan bukti (garis panduan, CPG, SOP, kajian saintifik) dengan pengambilan risiko apabila perlu.*  *(cth: Terdapat garis panduan untuk balutan luka, tetapi apabila situasi tidak mengizinkan, saya mengambil risiko dengan menggunakan teknik yang lain)* |
| J35 | I am committed to enhancing care by challenging practice | Words bearing emotional weight and action words | Same issue as item E17. No changes across 5 pretests. Main problem was the word ‘mempertikai’ (to challenge) | *Saya komited untuk meningkatkan kualiti jagaan dengan mempertikai amalan kerja sedia ada.* |
| K36 | I pay attention to the impact of the physical environment on people’s dignity | - | No Modification | *Saya memberi perhatian bagaimana persekitaran fizikal memberi kesan terhadap maruah individu (cth: kesan daripada perkongsian bilik konsultasi).* |
| K37 | I challenge others to consider how different elements of the physical environment impact on person-centredness (e.g. noise, light, heat etc) | Words bearing emotional weight and action words | Across all 5 pretests: Cannot translate ‘I challenge’ contextually to Malay language  The Malay word ‘mencabar’ carries the sole meaning of to dare someone, which was the wrong translation for the term ‘to challenge’. Challenge here refers to, to stimulate or trigger by the way of disputing someone, which has no direct translation in Malay.  Although the same word ‘challenge’ in item E17 was accurately represented by the word ‘mempertikai', in this item, the same word ‘mempertikai’ carries different meaning as the context is different.  Several terms were tried but none closely represent ‘to challenge’. Terms tried: *‘mencabar’, ‘menggalakkan’. ‘menggesa’*  Team discussed and decided to use the word *‘menggesa’* (to urge). It carries the closest meaning to the original question, although the term itself was not the right translation | *Saya menggesa orang lain untuk mempertimbangkan bagaimana unsur-unsur persekitaran mempengaruhi person-centredness (cth: bunyi bising, cahaya, kepanasan, dan lain-lain).* |
| K38 | I seek out creative ways of improving the physical environment | - | No Modification | *Saya mencari cara kreatif untuk memperbaiki persekitaran fizikal.* |
| L39 | In my team we take time to celebrate our achievements | - | No Modification | *Kami meluangkan masa untuk meraikan pencapaian dalam pasukan kami*. |
| L40 | My organization recognizes and rewards success | - | No Modification | *Organisasi saya mengenalpasti dan memberi ganjaran atas kejayaan.* |
| L41 | I am recognized for the contribution that I make to people having a good experience of care | Similar words with multiple meanings | PT1,2: Translated term for ‘recognised’*(dikenali)*  was misinterpreted into a different meaning.  PT3: The translated term was changed to another term which has a closer meaning to ‘being appreciated’ | *Saya dihargai atas sumbangan saya dalam memberikan pengalaman jagaan yang baik kepada individu.* |
| L42 | I am supported to express concerns about an aspect of care | - | No Modification | *Saya mendapat sokongan untuk menyuarakan rasa bimbang terhadap sebarang aspek jagaan.* |
| L43 | I have opportunity to discuss my practice and professional development on a regular basis | - | No Modification | *Saya berpeluang membincangkan perihal amalan kerja dan pembangunan kerjaya saya secara berkala.*  *(pembangunan kerjaya: peluang melanjutkan pelajaran, kenaikan pangkat, pengiktirafan)* |
| M44 | I integrate my knowledge of the person into care delivery | Complex sentence Sentence structure | PT1: Difficult to understand  PT2: Sentence structure changed  PT4: The term ‘of the person’ (*‘tentang individu’*) highlighted in bold to redirect the focus of the sentence | *Pengetahuan saya* ***tentang individu*** *diterapkan ke dalam penyampaian jagaan kesihatan.* |
| M45 | I work with the person within the context of their family and carers | - | No Modification | *Saya bekerjasama dengan individu, dengan mengambilkira keadaan keluarga dan penjaga mereka.* |
| M46 | I seek feedback on how people make sense of their care experience | Sentence structure | PT1: Focus of the question was deviated to getting general feedback, rather than feedback on patient’s experience  PT2: Sentence structure changed | *Saya mendapatkan maklumbalas pemahaman individu tentang apa yang dilaluinya semasa menerima jagaan.* |
| M47 | I encourage the people to discuss what is important to them | - | No Modification | *Saya menggalakkan individu untuk membincangkan apa yang penting bagi mereka.* |
| N48 | I include the family in care decisions where appropriate and/or in line with the person’s wishes | Sentence structure | PT1: Sentence too long  PT2: Sentence simplified | *Saya melibatkan ahli keluarga individu dalam menentukan keputusan jagaan apabila bersesuaian dengan kehendak individu.* |
| N49 | I work with the person to set health goals for their future | - | No Modification | *Saya bekerjasama dengan individu untuk menetapkan matlamat kesihatan mereka di masa hadapan.* |
| N50 | I enable people receiving care to seek information about their care from other healthcare professionals | Sentence structure | PT1,2: Focus of the question was deviated to helping people obtain information about their treatment, instead of getting information from different providers.  PT3: Sentence structure changed | *Saya membolehkan individu mendapatkan maklumat daripada anggota kesihatan yang lain tentang jagaan kesihatan mereka.* |
| O51 | I try to understand the person’s perspective | - | No Modification | *Saya cuba memahami pandangan seseorang individu.* |
| O52 | I seek to resolve issues when my goals for the person differ from their perspective | Grammatical meaning | PT1,2: The translated term of ‘perspective’ (*perspektif*) was not familiar among respondents  PT3: The translated term changed | *Apabila matlamat saya untuk individu tersebut berbeza dengan matlamat dirinya, saya akan berusaha untuk mencari penyelesaian.* |
| O53 | I engage people in care processes where appropriate | Sentence structure | PT1,2: Sentence is too long  PT3: Sentence simplified | *Saya melibatkan individu dalam proses jagaan mereka mengikut kesesuaian.* |
| P54 | I actively listen to people receiving care to identify unmet needs | - | No Modification | *Saya mendengar perihal individu yang menerima jagaan untuk mengenalpasti keperluan yang tidak dipenuhi.* |
| P55 | I gather additional information to help me support the people receiving care | - | No Modification | *Saya mendapatkan maklumat tambahan untuk membantu saya menyokong individu yang menerima jagaan.* |
| P56 | I ensure my full attention is focused on the person when I am with them | - | No Modification | *Saya memberi sepenuh perhatian kepada individu tersebut semasa saya bersama mereka.* |
| Q57 | I strive to gain a sense of the whole person | - | No Modification | *Saya berusaha untuk memahami individu secara menyeluruh.* |
| Q58 | I assess the needs of the person, taking account of all aspects of their lives | - | No Modification | *Saya menilai keperluan individu dengan mengambilkira semua aspek kehidupan individu tersebut.* |
| Q59 | I deliver care that takes account of the whole person | - | No Modification | *Saya memberi jagaan dengan mengambilkira individu tersebut secara menyeluruh.* |

*PT: Pre-test round
